# Racial differences in familiarity, interest, and use of integrative medicine among patients with breast cancer

**DOI:** 10.1007/s10549-024-07363-1

**Published:** 2024-05-15

**Authors:** Jincong Q. Freeman, Jori B. Sheade, Fangyuan Zhao, Olufunmilayo I. Olopade, Dezheng Huo, Rita Nanda

**Affiliations:** 1 Department of Public Health Sciences, The University of Chicago, Chicago, IL USA; 2 Cancer Prevention and Control Program, UChicago Medicine Comprehensive Cancer Center, Chicago, IL USA; 3 Center for Health and the Social Sciences, The University of Chicago, Chicago, IL USA; 4 Section of Hematology/Oncology, Department of Medicine, The University of Chicago, Chicago, IL USA; 5 Department of Hematology and Medical Oncology, Lake Forest Hospital Cancer Center, Northwestern Medicine, Lake Forest, IL USA; 6 Center for Clinical Cancer Genetics & Global Health, The University of Chicago, Chicago, IL USA; 7 Department of Medicine, The University of Chicago, 5841 S Maryland Ave, Chicago, IL 60637 USA

**Keywords:** Integrative medicine, Racial disparities, Breast cancer, ChiMEC

## Abstract

**Purpose:**

Integrative medicine (IM) has received the American Society of Clinical Oncology’s endorsement for managing cancer treatment-related side effects. Little is known about racial differences in familiarity, interest, and use of IM among patients with breast cancer.

**Methods:**

Patients with breast cancer enrolled in the Chicago Multiethnic Epidemiologic Breast Cancer Cohort were surveyed regarding familiarity, interest, and use of acupuncture, massage, meditation, music therapy, and yoga. Familiarity and interest, measured by a 5-point Likert scale, was modeled using proportional odds. Use was self-reported, and modeled using binary logistic regression.

**Results:**

Of 1,300 respondents (71.4% White and 21.9% Black), Black patients were less likely than White patients to be familiar with acupuncture (aOR 0.60, 95% CI 0.41–0.87); there were no racial differences in familiarity with massage, meditation, music therapy, and yoga. While there were no differences in interest in acupuncture between Black and White patients (aOR 1.12, 95% CI 0.76–1.65), Black patients were more interested in massage (aOR 1.86, 95% CI 1.25–2.77), meditation (aOR 2.03, 95% CI 1.37–3.00), music therapy (aOR 2.68, 95% CI 1.80–3.99), and yoga (aOR 2.10, 95% CI 1.41–3.12). Black patients were less likely than White patients to have used acupuncture (aOR 0.49, 95% CI 0.29–0.84); but there were no racial differences in use of massage, meditation, music therapy, and yoga.

**Conclusion:**

Black patients expressed more interest in IM than their White counterparts; there were no racial differences in IM use, except lower acupuncture use among Black patients. A breast program focused on equity should provide access to these services for patients with breast cancer.

## Introduction

In the United States (U.S.), breast cancer is the most common cancer type in women, accounting for approximately 30% of all cancers in women and with more than 3.6 million people alive with breast cancer in 2020 [1]. Breast cancer and its treatment can be associated with numerous side effects and symptoms, from cancer pain to lymphedema to hot flashes, [2] that can negatively impact patients’ treatment adherence and quality of life [3]. Reduction of side effects and management of symptoms typically consists of further medications, which carry their own adverse effect profiles. In 2017, the Society for Integrative Oncology (SIO) performed a systemic review focusing on randomized controlled trials from 1990 through 2015 of the use of integrative medicine (IM) modalities during and after breast cancer treatment [4]. This resulted in a set of evidence-based practice guidelines on the use of IM in breast cancer, which was subsequently endorsed by the American Society of Clinical Oncology (ASCO) in 2018 [5]. IM modalities, including acupuncture, therapeutic massage, meditation therapy, music therapy, and yoga therapy all have received ASCO’s endorsement for the treatment or management of various side effects and symptoms, particularly: hot flashes, nausea, anxiety or stress reduction, depression or mood disorders, and improving quality of life [5].

A 2021 study documented that more than 50.0% of cancer patients and their caregivers are familiar with acupuncture, yoga therapy, and meditation therapy [6]. Multiple previous studies have reported breast and gynecological cancer patients’ growing interest in and demand for IM, however, these studies included only White female patients [7–10]. Use of IM has increased in recent decades [11–13]. A 2005 survey conducted in breast cancer survivors found that more than 80.0% of the survivors had either used a complementary and alternative therapy or visited a therapist in the past [11]. According to a study using the 2002 National Health Interview Survey, Black race and lower socioeconomic status (SES) have historically been associated with lower prevalence of IM utilization among U.S. adults [12]. Therefore, IM services are generally marketed toward White populations and those with higher SES. However, little is known about familiarity, interest, and use of IM among Black or African American patients with breast cancer and survivors.

To date, the best study about racial differences in interest and use of IM comes from a 2017 study at the University of Texas MD Anderson Cancer Center, which surveyed 165 cancer patients, 43% of which were Black or African Americans, at an urban community hospital about interest and use of complementary and alternative therapies [14]. The study found that 90.6% of the patients were interested in therapeutic massage, followed by 72.7% in meditation therapy, 69.8% in yoga therapy, and 49.7% in acupuncture [14]. However, most were unprofessionally guided use, and both past and current IM use were low. In this study, 13.8% of Black patients had used yoga therapy as compared to 42.9% of Asian and 25.7% of White patients, with no significant differences among these racial groups based on a Pearson’s Chi-square test. Moreover, the study did not perform multivariable regression analyses due to the small sample size [14].

Prior studies are small and descriptive, with the majority of cancer patients and survivors being White. Additionally, there is paucity of data on racial differences in familiarity, interest, and use of IM specifically among patients with breast cancer. To fill these gaps in the literature, we sought to assess racial differences in familiarity, interest, and use of five ASCO-endorsed IM modalities for breast cancer symptom management: acupuncture, therapeutic massage, meditation therapy, music therapy, and yoga therapy in a large cohort of patients with breast cancer having been treated at the University of Chicago Medicine.

## Methods

### Study design and study population

We conducted a cross-sectional survey among patients with breast cancer who were enrolled in the Chicago Multiethnic Epidemiologic Breast Cancer Cohort (ChiMEC). Briefly, ChiMEC is a hospital-based study having been enrolling patients diagnosed with breast cancer since 1993. Detailed information of ChiMEC has been previously published [15]. Eligible participants were aged 18 years or older. From July to September 2021, a REDCap survey was sent to 2,788 ChiMEC participants who consented to be followed up for subsequent surveys. All patients provided their written informed consent prior to study participation. The University of Chicago Institutional Review Board reviewed and approved this study.

### Measures of key variables

Familiarity was measured by asking participants how familiar they were with these types of IM, using a 5-point Likert scale including not familiar at all, not very familiar, neutral, familiar, and very familiar. Interest was measured by asking participants how interested they would be in these IM modalities if offered at the center, using a 5-point Likert scale including not interested at all, not very interested, neutral, interested, and very interested.

We also assessed cancer treatment-related symptoms as facilitators by asking participants how interested they would be in any type of IM if it treated hot flashes, chemotherapy-induced neuropathy, nausea, joint pain, back or other pain, depression or mood change, fatigue or tiredness, and anxiety or stress reduction. Other facilitating factors for interest included recommendation from a provider, cost not being a barrier, being covered by health insurance, and price willing to pay out-of-pocket for a session.

To measure use of IM, we asked patients to “Select all of the therapies that you have had or received in the past.” Response options for each item were yes and no. We also assessed major barriers to IM use, including cost/money, lack of access to services, lack of transportation to service-providing facilities, lack of time, lack of interest, unaware of benefits of these services, low confidence about the benefits on these services, and lack of trusted information on these services by asking what, in general, prevents participants from using any IM modality.

### Covariates

Demographic and behavioral characteristics, including age, race/ethnicity (Asian, Black, Hispanic, and White), highest level of education (High school/GED or less, post high school, trade/technical school, or some college, Associate’s degree, Bachelor’s degree, and graduate or professional degree), marital status, annual household income, type of health insurance (Medicare, Medicaid, private, and other), and history of tobacco and alcohol consumption (never, current, and past), were collected from the survey. Clinical characteristics such as duration from diagnosis to survey, American Joint Committee on Cancer (AJCC) stage group, hormone receptor (HR) status (HR-positive/ human epidermal growth factor receptor 2 [HER2]-negative, HER2-positive, and triple negative breast cancer [TNBC]), and Charlson comorbidity index (CCI, i.e., 0, 1, and ≥ 2) was obtained through clinical chart abstraction.

### Statistical analysis

We calculated means and standard deviations (SD) for continuous data and tabulated frequencies and percentages (%) for categorical data. Demographic, behavioral, and clinical characteristics between racial/ethnic groups were compared using Student’s *t* tests or ANOVA for continuous variables and Pearson’s chi-square or Fisher’s exact tests for categorical variables. Of note, Asian and Hispanic patients were included in the descriptive analysis but were excluded in subsequent analyses due to small group sample size. Multivariable proportional odds were modeled for familiarity with and interest in different IM modalities. Multivariable binary logistic regression was modeled for self-report of having used these modalities in the past. All models were adjusted for age, highest level of education, marital status, annual household income, type of health insurance, CCI, HR status, and AJCC stage group. To assess racial differences in familiarity, interest, and use of IM modalities, we calculated adjusted odds ratios (aOR) and 95% confidence intervals (CI). The level of significance was set at 0.05. All statistical analyses were performed using SAS 9.4 (SAS Institute, Cary, NC).

## Results

### Patient characteristics

We received 1,300 survey responses from ChiMEC participants. Of the total, 71.4% were White, followed by 21.9% Black, 3.3% Asian, and 3.3% Hispanic; 59.1% were aged 40–65 years; 39.3% obtained a graduate or professional degree; 69.6% were married; 74.8% had private insurance and 23.5% were on Medicaid/Medicare; 65.8% were HR-positive/HER2-negative; and 98.9% had stage 0-III disease (Table 1). Given low percentages of Asian and Hispanic respondents, they were excluded in the following analyses. The mean age of White respondents was 61.4 (SD = 11.3) years, whereas Black respondents’ mean age was 62.6 (SD = 12.3) years. Compared with White patients, Black patients had higher percentages of having obtained high school/GED or less education (16.7% vs. 8.1%), a lower annual household income of less than $50,000 (43.4% vs. 11.3%), been enrolled in Medicaid (16.3% vs. 1.5%) or Medicare (25.2% vs. 17.2%), and a CCI of ≥ 2 (10.8% vs. 5.7%). A higher percentage of Black patients had TNBC than White patients (28.7% vs. 14.4%) (Table 1).

**Table 1:** Demographic, behavioral, and clinical characteristics of breast cancer patients, overall and by race/ethnicity

Characteristic	Total (*N* = 1300) *n* (%)	White (*N* = 928) *n* (%)	Black (*N* = 285) *n* (%)	Asian (*N* = 43) *n* (%)	Hispanic (*N* = 43) n (%)	*p* value^a^
Age^b^, mean (SD)	61.2 (11.7)	61.4 (11.3)	62.6 (12.3)	54.5 (12.5)	52.8 (11.1)	< 0.001
Age group^b^ (year)						
< 40	53 (4.1)	29 (3.1)	14 (4.9)	5 (11.6)	5 (11.6)	< 0.001
40–65	768 (59.1)	555 (59.8)	149 (52.3)	31 (72.1)	32 (74.4)	
> 65	479 (36.9)	344 (37.1)	122 (42.58)	7 (16.3)	6 (14.0)	
Highest level of education						
High school/GED or less	127 (10.2)	73 (8.1)	45 (16.7)	0	9 (23.1)	< 0.001
Post high school, trade/technical school, or some college	202 (16.2)	126 (14.0)	69 (25.7)	0	7 (18.0)	
Associate’s degree	87 (7.0)	58 (6.5)	21 (7.8)	4 (9.5)	4 (10.3)	
Bachelor’s degree	341 (27.3)	263 (29.3)	55 (20.5)	16 (38.1)	7 (18.0)	
Graduate or professional degree	491 (39.3)	378 (42.1)	79 (29.4)	22 (52.4)	12 (30.8)	
Marital status^c^						
Single or never married/unmarried or domestic partner	230 (19.7)	99 (11.8)	113 (45.0)	8 (20.0)	10 (24.4)	< 0.001
Married	814 (69.6)	661 (79.0)	96 (38.3)	29 (72.5)	28 (68.3)	
Separated/divorced	80 (6.8)	47 (5.6)	27 (10.8)	3 (7.5)	3 (7.3)	
Widowed	45 (3.9)	30 (3.6)	15 (6.0)	0	0	
Annual household income						
< $50,000	190 (20.0)	75 (11.3)	96 (43.4)	6 (18.8)	13 (39.4)	< 0.001
$50,000–$74,999	155 (16.3)	95 (14.3)	53 (24.0)	2 (6.3)	5 (15.2)	
$75,000–$99,999	128 (13.5)	84 (12.6)	34 (15.4)	7 (21.9)	3 (9.1)	
$100,000–$149,999	174 (18.3)	142 (21.4)	19 (8.6)	9 (28.1)	4 (12.1)	
$150,000–$199,999	131 (13.8)	111 (16.7)	13 (5.9)	2 (6.3)	5 (15.2)	
≥ $200,000	173 (18.2)	158 (23.8)	6 (2.7)	6 (18.8)	3 (9.1)	
Type of health insurance^d^						
Private	867 (74.8)	656 (79.6)	146 (56.6)	36 (92.3)	29 (76.3)	< 0.001
Medicaid	58 (5.0)	12 (1.5)	42 (16.3)	0	4 (10.5)	
Medicare	214 (18.5)	142 (17.2)	65 (25.2)	3 (7.7)	4 (10.5)	
Other	20 (1.7)	14 (1.7)	5 (1.9)	0	1 (2.6)	
History of tobacco use^e^						
Never	773 (68.4)	565 (70.5)	149 (60.1)	37 (92.5)	22 (53.7)	< 0.001
Current	70 (6.2)	41 (5.1)	23 (9.3)	0	6 (14.6)	
Past	288 (25.5)	196 (24.4)	76 (30.7)	3 (7.5)	13 (31.7)	
History of alcohol consumption						
Never	460 (41.5)	291 (36.7)	127 (53.4)	26 (68.4)	16 (41.0)	< 0.001
Current	626 (56.5)	488 (61.5)	105 (44.1)	12 (31.6)	21 (53.9)	
Past	23 (2.1)	15 (1.9)	6 (2.5)	0	2 (5.1)	
Duration from diagnosis to survey^f^ (year)						
Mean (SD)	7.2 (5.4)	7.2 (5.4)	7.8 (5.7)	6.0 (4.2)	4.8 (4.7)	0.004
Median (IQR)	6.0 (3.0–10.0)	6.0 (3.0–10.0)	6.0 (3.0–10.0)	5 (3.0–8.0)	4.0 (2.0–6.0)	0.001
Duration from diagnosis to survey^f^ (year)						
≤ 6	661 (53.2)	465 (52.4)	138 (50.6)	27 (64.3)	31 (75.6)	0.01
> 6	582 (46.8)	422 (47.6)	135 (49.5)	15 (35.7)	10 (24.4)	
Charlson comorbidity index						
0	1,033 (87.9)	746 (89.0)	214 (82.3)	35 (89.7)	38 (100.0)	0.018
1	63 (5.4)	44 (5.3)	18 (6.9)	1 (2.6)	0	
≥ 2	79 (6.7)	48 (5.7)	28 (10.8)	3 (7.7)	0	
Receptor status/subtype						
HR+/HER2-	607 (65.8)	455 (68.9)	111 (55.0)	18 (62.1)	23 (71.9)	< 0.001
HER2+	154 (16.7)	110 (16.7)	33 (16.3)	7 (24.1)	4 (12.5)	
TNBC	162 (17.6)	95 (14.4)	58 (28.7)	4 (13.8)	5 (15.6)	
Stage group^g^						
0	220 (18.0)	149 (17.1)	57 (21.1)	10 (23.8)	4 (10.3)	0.094
I	555 (45.3)	419 (48.0)	101 (37.4)	17 (40.5)	18 (46.2)	
II	314 (25.7)	216 (24.7)	76 (28.2)	9 (21.4)	13 (33.3)	
III	122 (10.0)	83 (9.5)	30 (11.1)	6 (14.3)	3 (7.7)	
IV	13 (1.1)	6 (0.7)	6 (2.2)	0	1 (2.6)	

SD, standard deviation; GED, general educational development; HR, hormone receptors; HER2, human epidermal growth factor receptor 2; TNBC, triple negative breast cancer; BCS, breast conserving surgery

Percentages may not be 100 due to rounding

^a^*p* values were calculated using ANOVA for continuous data and Chi-square or Fisher’s exact tests for categorical data

^b^Age was measured at the time of survey

^c^Marital status was documented at the time of diagnosis

^d^Other included 1 uninsured/self-pay, 17 insurance not otherwise specified, 1 TRICARE, and 1 Military

^e^Tobacco products assessed included cigarette, cigar, pipe, snuff, chew, smokeless, or mixed use with more than one type

^f^Duration from diagnosis to survey was defined as the duration from the time of cancer diagnosis to the time of survey

^g^Stage group was defined based on the American Joint Committee on Cancer’s cancer staging

### Familiarity with integrative medicine

Overall, 59.8% of the patients were familiar or very familiar with therapeutic massage, followed by 47.7% acupuncture, 47.6% meditation therapy, 47.0% yoga therapy, and 35.4% music therapy. Compared with White patients, Black patients had a higher percentage of being familiar or very familiar with music therapy (44.2% vs. 32.7%). However, a higher percentage of White patients reported being familiar or very familiar with therapeutic massage (60.9% vs. 56.4%), acupuncture (49.9% vs. 40.5%), or yoga therapy (47.8% vs. 44.3%) than Black patients (Table 2). After adjusting for covariates, Black patients were less likely than their White counterparts to be familiar with acupuncture (aOR 0.60, 95% CI 0.41–0.87). We did not observe differences between Black and White patients in familiarity with music therapy, meditation therapy, therapeutic, or yoga therapy (Table 3).

**Table 2:** Familiarity with integrative medicine modalities among breast cancer patients, overall and by race

Type of service	Familiarity^a^	Total (*N* = 1213)	White (*N* = 928)	Black (*N* = 285)	*p* value^b^
		n (%)	n (%)	n (%)	
Acupuncture	Not familiar at all (1)	254 (21.2)	169 (18.4)	85 (30.5)	< 0.001
	Not very familiar (2)	227 (18.9)	177 (19.2)	50 (17.9)	
	Neutral (3)	147 (12.3)	116 (12.6)	31 (11.1)	
	Familiar (4)	356 (29.7)	287 (31.2)	69 (24.7)	
	Very familiar (5)	216 (18.0)	172 (18.7)	44 (15.8)	
	Mean (SD)	3.0 (1.4))	3.1 (1.4)	2.8 (1.5)	< 0.001
Therapeutic massage	Not familiar at all (1)	170 (14.2)	113 (12.3)	57 (20.4)	0.012
	Not very familiar (2)	175 (14.6)	134 (14.6)	41 (14.6)	
	Neutral (3)	135 (11.3)	111 (12.1)	24 (8.6)	
	Familiar (4)	444 (37.1)	347 (37.9)	97 (34.6)	
	Very familiar (5)	272 (22.7)	211 (23.0)	61 (21.8)	
	Mean (SD)	3.4 (1.4)	3.4 (1.3)	3.2 (1.5)	0.026
Meditation therapy	Not familiar at all (1)	223 (18.7)	155 (16.9)	68 (24.6)	0.009
	Not very familiar (2)	210 (17.6)	163 (17.8)	47 (17.0)	
	Neutral (3)	193 (16.2)	163 (17.8)	30 (10.8)	
	Familiar (4)	389 (32.6)	302 (32.9)	87 (31.4)	
	Very familiar (5)	179 (15.0)	134 (14.6)	45 (16.3)	
	Mean (SD)	3.1 (1.4)	3.1 (1.3)	3.0 (1.5)	0.194
Music therapy	Not familiar at all (1)	284 (24.0)	217 (23.9)	67 (24.3)	0.001
	Not very familiar (2)	269 (22.7)	214 (23.5)	55 (19.9)	
	Neutral (3)	213 (18.0)	181 (19.9)	32 (11.6)	
	Familiar (4)	308 (26.0)	221 (24.3)	87 (31.5)	
	Very familiar (5)	111 (9.4)	76 (8.4)	35 (12.7)	
	Mean (SD)	2.7 (1.3)	2.7 (1.3)	2.9 (1.4)	0.04
Yoga therapy	Not familiar at all (1)	236 (20.1)	161 (17.9)	75 (27.5)	< 0.001
	Not very familiar (2)	194 (16.6)	141 (15.7)	53 (19.4)	
	Neutral (3)	191 (16.3)	167 (18.6)	24 (8.8)	
	Familiar (4)	374 (31.9)	294 (32.7)	80 (29.3)	
	Very familiar (5)	177 (15.1)	136 (15.1)	41 (15.0)	
	Mean (SD)	3.1 (1.4)	3.1 (1.3)	2.8 (1.5)	0.008

*SD* standard deviation

Percentages may not be 100 due to rounding

^a^Familiarity was assessed by asking participants how familiar they were with integrative medicine modalities

^b^*p* values were calculated using Student’s t tests for continuous data and Chi-square tests for categorical data

**Table 3:** Multivariable regression models for familiarity, interest, and self-reported use of different integrative medicine modalities among breast cancer patients, comparing Black patients with White patients

Type of service	Familiarity^a^		Interest^b^		Self-reported use^c^	
	aOR^d^ (95% CI)	*p* value	aOR^d^ (95% CI)	*p* value	aOR^d^ (95% CI)	*p* value
Acupuncture	0.60 (0.41–0.87)	0.007	1.12 (0.76–1.65)	0.568	0.49 (0.29–0.84)	0.009
Therapeutic massage	0.91 (0.63–1.33)	0.638	1.86 (1.25–2.77)	0.002	0.83 (0.53–1.30)	0.413
Meditation therapy	1.01 (0.69–1.46)	0.982	2.03 (1.37–3.00)	< 0.001	0.82 (0.47–1.43)	0.483
Music therapy	1.26 (0.87–1.84)	0.226	2.68 (1.80–3.99)	< 0.001	1.65 (0.82–3.32)	0.164
Yoga therapy	0.71 (0.49–1.04)	0.077	2.10 (1.41–3.12)	< 0.001	0.67 (0.37–1.20)	0.179

*aOR* adjusted odds ratio, *CI* confidence interval

Multivariable proportional odds were modeled for familiarity with and interest in using different integrative medicine (IM) modalities, while multivariable binomial logistic regression was modeled for self-report of having used these IM modalities in the past

^a^Familiarity was assessed by asking participants how familiar they were with these IM modalities

^b^Interest was assessed by asking participants how interested they would be in these IM modalities if offered at the center

^c^Participants were asked about these IM modalities they have used in the past

^d^Adjusted for age, highest level of education, marital status, annual household income, type of health insurance, Charlson comorbidity index, hormone receptor status/subtype, and stage group

### Interest in integrative medicine and facilitators

Overall, 62.9% of the patients reported being interested or very interested in therapeutic massage, followed by 49.4% yoga therapy, 47.1% meditation therapy, 43.9% acupuncture, and 40.4% music therapy. By race, Black patients had higher percentages of being interested or very interested in therapeutic massage (74.0% vs. 59.5%), yoga therapy (62.6% vs. 45.5%), meditation therapy (60.7% vs. 43.1%), music therapy (56.2% vs. 35.8%), or acupuncture (49.2% vs. 42.3%) than did White patients (Table 4). In the adjusted proportional odds model, Black patients were significantly more interested in the use of music therapy (aOR 2.68, 95% CI 1.80–3.99), yoga therapy (aOR 2.10, 95% CI 1.41–3.12), meditation therapy (aOR 2.03, 95% CI 1.37–3.00), and therapeutic massage (aOR 1.86, 95% CI 1.25–2.77) than their White counterparts. There were no differences in interest in acupuncture between the racial groups (Table 3).

**Table 4:** Interest in using integrative medicine modalities among breast cancer patients, overall and by race

Type of service	Interest^a^	Total (*N* = 1213)	White (*N* = 928)	Black (*N* = 285)	*p* value^b^
		*n* (%)	*n* (%)	*n* (%)	
Acupuncture	Not interested at all (1)	300 (26.3)	231 (26.1)	69 (27.2)	0.151
	Not very interested (2)	143 (12.5)	116 (13.1)	27 (10.6)	
	Neutral (3)	197 (17.3)	164 (18.5)	33 (13.0)	
	Interested (4)	245 (21.5)	185 (20.9)	60 (23.6)	
	Very interested (5)	255 (22.4)	190 (21.4)	65 (25.6)	
	Mean (SD)	3.0 (1.5)	3.0 (1.5)	3.1 (1.6)	0.294
Therapeutic massage	Not interested at all (1)	196 (16.8)	165 (18.4)	31 (11.5)	0.001
	Not very interested (2)	80 (6.9)	67 (7.5)	13 (4.8)	
	Neutral (3)	157 (13.5)	131 (14.6)	26 (9.7)	
	Interested (4)	339 (29.1)	246 (27.4)	93 (34.6)	
	Very interested (5)	394 (33.8)	288 (32.1)	106 (39.4)	
	Mean (SD)	3.6 (1.4)	3.5 (1.5)	3.9 (1.3)	< 0.001
Meditation therapy	Not interested at all (1)	243 (21.5)	203 (23.3)	40 (15.6)	< 0.001
	Not very interested (2)	131 (11.6)	109 (12.5)	22 (8.6)	
	Neutral (3)	224 (19.8)	185 (21.2)	39 (15.2)	
	Interested (4)	284 (25.1)	204 (23.4)	80 (31.1)	
	Very interested (5)	248 (22.0)	172 (19.7)	76 (29.6)	
	Mean (SD)	3.1 (1.4)	3.0 (1.4)	3.5 (1.4)	< 0.001
Music therapy	Not interested at all (1)	252 (22.7)	218 (25.4)	34 (13.6)	< 0.001
	Not very interested (2)	150 (13.5)	130 (15.2)	20 (8.0)	
	Neutral (3)	259 (23.4)	203 (23.7)	56 (22.3)	
	Interested (4)	252 (22.7)	181 (21.1)	71 (28.3)	
	Very interested (5)	196 (17.7)	126 (14.7)	70 (27.9)	
	Mean (SD)	3.0 (1.4)	2.8 (1.4)	3.5 (1.3)	< 0.001
Yoga therapy	Not interested at all (1)	250 (22.2)	205 (23.4)	45 (17.9)	< 0.001
	Not very interested (2)	111 (9.8)	94 (10.7)	17 (6.8)	
	Neutral (3)	210 (18.6)	178 (20.3)	32 (12.7)	
	Interested (4)	294 (26.1)	213 (24.3)	81 (32.0)	
	Very interested (5)	263 (23.3)	186 (21.2)	77 (30.6)	
	Mean (SD)	3.2 (1.5)	3.1 (1.5)	3.5 (1.4)	< 0.001

*SD* standard deviation

Percentages may not be 100 due to rounding

^a^Interest was assessed by asking participants how interested they would be in these integrative medicine modalities if offered at the center

^b^*p* values were calculated using Student’s t tests for continuous data and Chi-square tests for categorical data

Participants reported being interested or very interested when asked for interest in any of the IM modalities to address specific symptoms: joint paints due to aromatase inhibitors (Black 71.6% vs. White 66.2%), back pain or other pain (Black 71.0% vs. White 65.9%), fatigue (Black 63.8% vs. White 64.0%), anxiety or stress reduction (Black 61.3% vs. White 63.25), depression or mood changes (Black 50.4% vs. White 52.5%), hot flashes (Black 49.2% vs. White 44.1%), chemotherapy-induced neuropathy (Black 43.2% vs. White 38.0%), and nausea (Black 32.0% vs. White 30.2%). However, there were no significant differences in symptoms as facilitators for interest in IM between the racial groups (Table 5). Generally, Black respondents expressed more interest in IM modalities if they were recommended by their doctors or nurses and were covered by health insurance. Black respondents typically were less willing to pay more than $0–$19 out of pocket for a session of any IM services (Table 6).

**Table 5:** Symptoms as facilitators for interest in using any integrative medicine modality among breast cancer patients, overall and by race

Symptoms as facilitators^a^	Total (*N* = 1213) *n* (%)	White (*N* = 928) *n* (%)	Black (*N* = 285) *n* (%)	*p* value^b^
Hot flashes				
Not interested (1,2)	410 (36.9)	330 (38.1)	80 (32.5)	0.254
Neutral (3)	199 (17.9)	154 (17.8)	45 (18.3)	
Interested (4,5)	503 (45.2)	382 (44.1)	121 (49.2)	
Chemotherapy-induced neuropathy				
Not interested (1,2)	453 (41.4)	354 (41.9)	99 (39.6)	0.297
Neutral (3)	213 (19.5)	170 (20.1)	43 (17.2)	
Interested (4,5)	429 (39.2)	321 (38.0)	108 (43.2)	
Nausea				
Not interested (1,2)	487 (45.3)	375 (45.0)	112 (46.5)	0.579
Neutral (3)	259 (24.1)	207 (24.8)	52 (21.6)	
Interested (4,5)	329 (30.6)	252 (30.2)	77 (32.0)	
Joint pain				
Not interested (1,2)	223 (19.4)	175 (20.0)	48 (17.7)	0.227
Neutral (3)	150 (13.1)	121 (13.8)	29 (10.7)	
Interested (4,5)	774 (67.5)	580 (66.2)	194 (71.6)	
Back or other pain				
Not interested (1,2)	221 (19.5)	169 (19.5)	52 (19.3)	0.108
Neutral (3)	152 (13.4)	126 (14.6)	26 (9.7)	
Interested (4,5)	762 (67.1)	571 (65.9)	191 (71.0)	
Depression/mood change				
Not interested (1,2)	319 (28.8)	238 (27.7)	81 (32.4)	0.313
Neutral (3)	213 (19.2)	170 (19.8)	43 (17.2)	
Interested (4,5)	577 (52.0)	451 (52.5)	126 (50.4)	
Fatigue/tiredness				
Not interested (1,2)	245 (21.6)	184 (21.2)	61 (23.0)	0.705
Neutral (3)	164 (14.5)	129 (14.8)	35 (13.2)	
Interested (4,5)	726 (64.0)	557 (64.0)	169 (63.8)	
Anxiety/stress reduction				
Not interested (1,2)	248 (22.3)	192 (22.3)	56 (22.1)	0.711
Neutral (3)	167 (15.0)	125 (14.5)	42 (16.6)	
Interested (4,5)	699 (62.8)	544 (63.2)	155 (61.3)	

Percentages may not be 100 due to rounding

^a^Symptoms as facilitators were assessed by asking participants how interested they would be in any integrative medicine modality if specifically treated the symptoms above

^b^*p* values were calculated using Chi-square tests

**Table 6:** Facilitators for interest in using integrative medicine modalities among breast cancer patients, overall and by race

Type of service	Facilitator	Total (*N* = 1213) *n *(%)	White (*N* = 928) *n* (%)	Black (*N* = 285) *n* (%)	*p* value^a^
Acupuncture	Recommendation from your doctor or nurse^b^				
	Not interested at all (1)	206 (18.1)	141 (16.0)	65 (25.1)	0.017
	Not very interested (2)	101 (8.9)	79 (9.0)	22 (8.5)	
	Neutral (3)	186 (16.3)	152 (17.2)	34 (13.1)	
	Interested (4)	320 (28.1)	252 (28.6)	68 (26.3)	
	Very interested (5)	328 (28.8)	258 (29.3)	70 (27.0)	
	Cost not being a barrier^c^				
	Not interested at all (1)	201 (18.0)	141 (16.3)	60 (23.5)	0.073
	Not very interested (2)	96 (8.6)	74 (8.6)	22 (8.6)	
	Neutral (3)	182 (16.3)	149 (17.3)	33 (12.9)	
	Interested (4)	261 (23.3)	201 (23.3)	60 (23.5)	
	Very interested (5)	379 (33.9)	299 (34.6)	80 (31.4)	
	Being covered by health insurance^d^				
	Not interested at all (1)	207 (18.4)	145 (16.7)	62 (24.2)	0.065
	Not very interested (2)	90 (8.0)	68 (7.8)	22 (8.6)	
	Neutral (3)	160 (14.2)	130 (15.0)	30 (11.7)	
	Interested (4)	267 (23.8)	207 (23.9)	60 (23.4)	
	Very interested (5)	400 (35.6)	318 (36.6)	82 (32.0)	
	Price willing to pay out of pocket for a session				
	$0	433 (40.1)	303 (36.4)	130 (52.2)	< 0.001
	$1–$19	152 (14.1)	116 (13.9)	36 (14.5)	
	$20–$39	216 (20.0)	183 (22.0)	33 (13.3)	
	$40–$59	162 (15.0)	131 (15.8)	31 (12.5)	
	$60–$79	52 (4.8)	43 (5.2)	9 (3.6)	
	$80–$99	36 (3.3)	33 (4.0)	3 (1.2)	
	$100+	30 (2.8)	23 (2.8)	7 (2.8)	
Therapeutic massage	Recommendation from your doctor or nurse^b^				
	Not interested at all (1)	106 (9.1)	80 (8.9)	26 (9.5)	0.402
	Not very interested (2)	46 (3.9)	34 (3.8)	12 (4.4)	
	Neutral (3)	147 (12.6)	122 (13.6)	25 (9.2)	
	Interested (4)	414 (35.4)	317 (35.3)	97 (35.5)	
	Very interested (5)	458 (39.1)	345 (38.4)	113 (41.4)	
	Cost not being a barrier^c^				
	Not interested at all (1)	104 (9.1)	79 (9.0)	25 (9.4)	0.344
	Not very interested (2)	50 (4.4)	41 (4.7)	9 (3.4)	
	Neutral (3)	141 (12.3)	116 (13.2)	25 (9.4)	
	Interested (4)	337 (29.5)	250 (28.5)	87 (32.6)	
	Very interested (5)	512 (44.8)	391 (44.6)	121 (45.3)	
	Being covered by health insurance^d^				
	Not interested at all (1)	103 (8.9)	79 (9.0)	24 (8.8)	0.414
	Not very interested (2)	49 (4.3)	38 (4.3)	11 (4.0)	
	Neutral (3)	123 (10.7)	101 (11.5)	22 (8.0)	
	Interested (4)	346 (30.0)	254 (28.9)	92 (33.6)	
	Very interested (5)	531 (46.1)	406 (46.2)	125 (45.6)	
	Price willing to pay out of pocket for a session				
	$0	268 (24.2)	178 (21.1)	90 (34.2)	< 0.001
	$1–$19	179 (16.2)	128 (15.2)	51 (19.4)	
	$20–$39	267 (24.1)	219 (25.9)	48 (18.3)	
	$40–$59	198 (17.9)	159 (18.8)	39 (14.8)	
	$60–$79	103 (9.3)	90 (10.7)	13 (4.9)	
	$80–$99	52 (4.7)	41 (4.9)	11 (4.2)	
	$100+	41 (3.7)	30 (3.6)	11 (4.2)	
Meditation therapy	Recommendation from your doctor or nurse^b^				
	Not interested at all (1)	162 (14.2)	123 (14.0)	39 (15.0)	0.002
	Not very interested (2)	97 (8.5)	81 (9.2)	16 (6.2)	
	Neutral (3)	220 (19.3)	187 (21.3)	33 (12.7)	
	Interested (4)	354 (31.1)	271 (30.8)	83 (31.9)	
	Very interested (5)	307 (26.9)	218 (24.8)	89 (34.2)	
	Cost not being a barrier^c^				
	Not interested at all (1)	163 (14.7)	125 (14.6)	38 (14.8)	0.005
	Not very interested (2)	86 (7.7)	71 (8.3)	15 (5.8)	
	Neutral (3)	212 (19.1)	180 (21.1)	32 (12.5)	
	Interested (4)	298 (26.8)	226 (26.4)	72 (28.0)	
	Very interested (5)	353 (31.7)	253 (29.6)	100 (38.9)	
	Being covered by health insurance^d^				
	Not interested at all (1)	161 (14.3)	124 (14.3)	37 (14.3)	0.001
	Not very interested (2)	86 (7.7)	73 (8.4)	13 (5.0)	
	Neutral (3)	202 (18.0)	175 (20.2)	27 (10.5)	
	Interested (4)	305 (27.2)	228 (26.4)	77 (29.8)	
	Very interested (5)	369 (32.9)	265 (30.6)	104 (40.3)	
	Price willing to pay out of pocket for a session				
	$0	446 (41.8)	327 (40.1)	119 (47.2)	0.128
	$1–$19	224 (21.0)	178 (21.8)	46 (18.3)	
	$20–$39	222 (20.8)	177 (21.7)	45 (17.9)	
	$40–$59	116 (10.9)	92 (11.3)	24 (9.5)	
	$60–$79	25 (2.3)	19 (2.3)	6 (2.4)	
	$80-$99	19 (1.8)	14 (1.7)	5 (2.0)	
	$100+	15 (1.4)	8 (1.0)	7 (2.8)	
Music therapy	Recommendation from your doctor or nurse^b^				
	Not interested at all (1)	177 (15.9)	139 (16.2)	38 (15.0)	< 0.001
	Not very interested (2)	117 (10.5)	103 (12.0)	14 (5.5)	
	Neutral (3)	251 (22.6)	206 (24.0)	45 (17.7)	
	Interested (4)	314 (28.2)	233 (27.1)	81 (31.9)	
	Very interested (5)	254 (22.8)	178 (20.7)	76 (29.9)	
	Cost not being a barrier^c^				
	Not interested at all (1)	177 (16.2)	143 (17.0)	34 (13.6)	< 0.001
	Not very interested (2)	109 (10.0)	98 (11.7)	11 (4.4)	
	Neutral (3)	241 (22.1)	198 (23.5)	43 (17.2)	
	Interested (4)	264 (24.2)	196 (23.3)	68 (27.2)	
	Very interested (5)	300 (27.5)	206 (24.5)	94 (37.6)	
	Being covered by health insurance^d^				
	Not interested at all (1)	176 (16.1)	143 (17.0)	33 (13.2)	< 0.001
	Not very interested (2)	104 (9.5)	94 (11.2)	10 (4.0)	
	Neutral (3)	234 (21.5)	194 (23.1)	40 (15.9)	
	Interested (4)	281 (25.8)	207 (24.6)	74 (29.5)	
	Very interested (5)	296 (27.1)	202 (24.1)	94 (37.5)	
	Price willing to pay out of pocket for a session				
	$0	531 (50.7)	403 (50.3)	128 (52.0)	0.756
	$1–$19	198 (18.9)	152 (19.0)	46 (18.7)	
	$20–$39	176 (16.8)	140 (17.5)	36 (14.6)	
	$40–$59	96 (9.2)	74 (9.2)	22 (8.9)	
	$60–$79	23 (2.2)	17 (2.1)	6 (2.4)	
	$80–$99	9 (0.9)	7 (0.9)	2 (0.8)	
	$100+	15 (1.4)	9 (1.1)	6 (2.4)	
Yoga therapy	Recommendation from your doctor or nurse^b^				
	Not interested at all (1)	176 (15.6)	132 (15.1)	44 (17.3)	0.009
	Not very interested (2)	78 (6.9)	68 (7.8)	10 (3.9)	
	Neutral (3)	201 (17.8)	170 (19.5)	31 (12.2)	
	Interested (4)	373 (33.1)	281 (32.2)	92 (36.1)	
	Very interested (5)	300 (26.6)	222 (25.4)	78 (30.6)	
	Cost not being a barrier^c^				
	Not interested at all (1)	163 (14.9)	123 (14.5)	40 (16.2)	0.004
	Not very interested (2)	80 (7.3)	69 (8.1)	11 (4.5)	
	Neutral (3)	197 (18.0)	169 (19.9)	28 (11.3)	
	Interested (4)	296 (27.0)	221 (26.0)	75 (30.4)	
	Very interested (5)	361 (32.9)	268 (31.5)	93 (37.7)	
	Being covered by health insurance^d^				
	Not interested at all (1)	175 (15.7)	133 (15.5)	42 (16.3)	0.002
	Not very interested (2)	71 (6.4)	62 (7.2)	9 (3.5)	
	Neutral (3)	186 (16.7)	160 (18.6)	26 (10.1)	
	Interested (4)	302 (27.0)	222 (25.8)	80 (31.1)	
	Very interested (5)	383 (34.3)	283 (32.9)	100 (38.9)	
	Price willing to pay out of pocket for a session				
	$0	404 (37.7)	295 (35.8)	109 (44.1)	0.298
	$1–$19	258 (24.1)	209 (25.4)	49 (19.8)	
	$20–$39	247 (23.1)	196 (23.8)	51 (20.7)	
	$40–$59	101 (9.4)	77 (9.3)	24 (9.7)	
	$60–$79	31 (2.9)	24 (2.9)	7 (2.8)	
	$80–$99	12 (1.1)	10 (1.2)	2 (0.8)	
	$100+	18 (1.7)	13 (1.6)	5 (2.0)	

Percentages may not be 100 due to rounding

^a^*p* values were calculated using Chi-square tests

^b^Recommendation from your doctor or nurse was assessed by asking participants how interested they would be to have any integrative medicine modality if their doctor or nurse recommended it

^c^Cost not being a barrier was assessed by asking participants how interested they would be to have any integrative medicine modality if cost was not a barrier

^d^Being covered by health insurance was assessed by asking participants how interested they would be to have any integrative medicine modality if their health insurance paid for it

### Use of integrative medicine and barriers

Overall, 41.6% of the patients had used therapeutic massage, followed by 26.1% acupuncture, 19.0% yoga therapy, 18.5% meditation therapy, and only 7.7% music therapy. Higher percentages of White patients reported having used therapeutic massage (42.9% vs. 37.5%), acupuncture (29.5% vs. 14.7%), yoga therapy (20.8% vs. 13.0%), and meditation therapy (18.8% vs. 17.5%) than Black patients. However, Black patients had a higher percentage of prior use of music therapy than did White patients (11.2% vs. 6.6%) (Table 7). In the adjusted logistic regression model, there were no differences between Black and White patients in self-reported use of the IM modalities surveyed, with the exception of acupuncture as Black patients were less likely than their White counterparts to have used acupuncture (aOR 0.49, 95% CI 0.29–0.84) (Table 3).

**Table 7:** Self-reported use of integrative medicine modalities among breast cancer patients, overall and by race

Type of service	Total (*N* = 1213)	White (*N* = 928)	Black (*N* = 285)	*p* value^a^
	*n* (%)	*n* (%)	*n* (%)	
Acupuncture				
No	897 (73.9)	654 (70.5)	243 (85.3)	< 0.001
Yes	316 (26.1)	274 (29.5)	42 (14.7)	
Therapeutic massage				
No	708 (58.4)	530 (57.1)	178 (62.5)	0.109
Yes	505 (41.6)	398 (42.9)	107 (37.5)	
Meditation therapy				
No	989 (81.5)	754 (81.3)	235 (82.5)	0.646
Yes	224 (18.5)	174 (18.8)	50 (17.5)	
Music therapy				
No	1,120 (92.3)	867 (93.4)	253 (88.8)	0.009
Yes	93 (7.7)	61 (6.6)	32 (11.2)	
Yoga therapy				
No	983 (81.0)	735 (79.2)	248 (87.0)	0.003
Yes	230 (19.0)	193 (20.8)	37 (13.0)	

Percentages may not be 100 due to rounding

^a^*p* values were calculated using Chi-square tests

^b^Participants were asked about these integrative medicine modalities they have used in the past

When participants were asked about barriers to use of any IM modality, higher percentages of Black patients reported cost (55.1% vs. 31.4%), lack of awareness of benefits of IM services (35.8% vs. 23.9%), lack of access to services (24.6% vs. 19.4%), and lack of transportation to service-providing facilities (13.0% vs. 9.3%) than White patients. White patients had a higher percentage of lack of time as a barrier to use than their Black counterparts (32.2% vs. 19.3%). Of note, confidence in the benefits of IM was high in both races (White 91% vs. Black 90.5%) (Table 8).

**Table 8:** Barriers to using any integrative medicine modality among breast cancer patients, overall and by race

Barrier^a^	Total (*N* = 1213) *n* (%)	White (*N* = 928) *n* (%)	Black (*N* = 285) *n* (%)	*p* value^b^
Cost/money				
No	765 (63.1)	637 (68.6)	128 (44.9)	< 0.001
Yes	448 (36.9)	291 (31.4)	157 (55.1)	
Lack of access to services				
No	963 (79.4)	748 (80.6)	215 (75.4)	0.059
Yes	250 (20.6)	180 (19.4)	70 (24.6)	
Lack of transportation to service-providing facilities				
No	1,090 (89.9)	842 (90.7)	248 (87.0)	0.069
Yes	123 (10.1)	86 (9.3)	37 (13.0)	
Lack of time				
No	859 (70.8)	629 (67.8)	230 (80.7)	< 0.001
Yes	354 (29.2)	299 (32.2)	55 (19.3)	
Lack of interest				
No	1,017 (83.8)	768 (82.8)	249 (87.4)	0.064
Yes	196 (16.2)	160 (17.2)	36 (12.6)	

Percentages may not be 100 due to rounding

^a^Barriers were assessed by asking participants what, in general, prevents them from using any integrative medicine modality

^b^*p* values were calculated using Chi-square tests

## Discussion

To our knowledge, this is of the first and the largest study to examine racial differences in familiarity, interest, and use of five ASCO-endorsed IM modalities and to assess specific symptom-related facilitating factors for interest in, and key barriers to use of, these modalities among U.S. patients with breast cancer. In this diverse cohort of patients with breast cancer, familiarity of IM modalities was prevalent among Black and White patients. However, higher proportions, ranging from 26.9% to 48.6%, of the patients across the racial groups were still not familiar with these modalities. Black patients were less likely to be familiar with acupuncture than their White counterparts, but there were no differences between them in familiarity with therapeutic massage, meditation therapy, music therapy, and yoga therapy. These findings are somewhat consistent with a recent study of familiarity and interest in IM among cancer patients and their caregivers that non-White patients are less familiar with therapeutic massage than White patients, while levels of familiarity with acupuncture, meditation therapy, music therapy, and yoga therapy are similar between White and non-White patients [6]. Our findings suggest that patient education on IM and its associated benefits may be needed among patients with breast cancer in order to increase patients’ knowledge and awareness of IM. Future research may be needed to explore reasons related to level of familiarity and how the findings could help inform and tailor IM education campaigns and programs specifically toward patients with breast cancer and survivors.

We found that most patients across the two racial groups were interested in the use of IM modalities, and the percentages of interest of IM use also increased when the patients were asked if any of these modalities were treated for a particular symptom such as joint pains, back pain, fatigue, anxiety or stress reduction, hot flashes, and chemotherapy-induced neuropathy. Our finding in increased interest of IM use aligns with a published study that patients who experience back, joint, or other pain are more likely to use acupuncture and therapeutic massage, though racial differences were not assessed [16]. Further, Black patients were twice as likely as their White counterparts to have expressed interest in music therapy, yoga therapy, meditation therapy, and therapeutic massage, which is contrary to prior findings in the general population that conclude Black patients are less interested in IM services than White patients [12]. Patients with breast cancer may have unique needs, as a population-based research has indicated that cancer patients and survivors are more likely than the general population to have discussed IM use with a provider and have used these modalities in the past 12 months [17]. Our result also contradicts a recent study finding that levels of interest in IM are similar between non-White and White patients [6]. However, this study sample was relatively small, probably lacking statistical power. In addition, it included patients with different cancer types and their caregivers, and thus, the findings may not be comparable to our cohort of patients with breast cancer [6]. It is worth noting that approximately 21.6%–40.6% of the patients across the racial groups reported being not very interested or not interested at all in using these IM modalities, even when they were asked if these modalities addressed specific common cancer treatment-associated symptoms. It is also important to note that most patients expressed greater interest in IM use if recommended by their providers and were willing to pay no more than $19 out of pocket for a session. Our findings indicate that Black patients may be in greater need for IM and that both provision and coverage of these modalities should be integrated as part of standard cancer care and services at comprehensive cancer centers in the U.S.

The percentages of past use of IM modalities were low among Black and White patients with breast cancer; the majority of the patients, between 57.1% and 93.4%, had not used these modalities in the past. These results are in line with a previous study that both past and current use of acupuncture, meditation therapy, yoga therapy, and therapeutic massage were low, ranging from only 5.6% to 46.3% [14]. Compared with White patients, a higher proportion of Black patients reported cost, lack of access to services, unaware of the benefits of these services as major barriers to IM use. Less than 10.0% of both Black and White patients reported low confidence in the benefits of the five surveyed IM modalities. After adjusting for key demographic and clinical characteristics, Black patients in our cohort were significantly less likely than their White counterparts to have used acupuncture, while there were no differences between the racial groups in past use of therapeutic massage, meditation therapy, music therapy, or yoga therapy. Our finding is consistent with the previous study partially that use of yoga therapy was similar between Black and White patients, however, multivariable regression modeling was not performed due to the small sample size [14]. It is important to point out that we did not ask whether the patients had used these IM modalities before, during, and/or after their cancer therapies, which is worth doing in future research to evaluate whether there are racial and ethnic differences in IM utilization over time and how these differences would impact patients’ treatment adherence and quality of life. Our findings also suggest that providers at cancer centers should be promoting these IM services as recommended by ASCO guidelines to all patients which, as shown, may be likely to increase interest in patients with breast cancer and survivors.

Furthermore, Black patients with breast cancer more frequently report nonadherence to endocrine therapy than their White counterparts, with side effect profile being one of the main causes of discontinuation [18, 19]. Therefore, the use of IM modalities to reduce side effects from breast cancer treatment and to manage symptoms may lead to greater adherence to endocrine therapy in Black patients. As we have shown Black patients are just as interested, if not more interested, in IM as their White counterparts, and there are likely unmet needs of IM among patients with breast cancer, we should ensure equity to access these services for all our patients, regardless of race.

Several limitations of this study need to be noted. First, the data collected through the survey were self-reported, which were subject to recall error or social desirability bias. However, we expect such bias to be minimal since our research staff had little interaction with the patients. Therefore, their responses were unlikely influenced. Second, because we did not ask the participants whether they had use these services before their cancer diagnoses, during, and/or after their cancer treatment, with an approximately 47.0% response rate, the percentages of use of these IM modalities may have been either overestimated or underestimated. Third, we were not able to assess unmeasured characteristics, e.g., cultural background/influence, employment status, patient-provider discussion of IM, which might affect or help better explain the observed racial differences in familiarity, interest, and past use of IM. Thus, additional cultural and behavioral factors should be taken into consideration in future research. Lastly, participants in the ChiMEC may not be representative of all U.S. patients with breast cancer or other patient populations, and therefore, limiting the generalizability of our study findings.

Despite the above limitations, this study has several strengths. Our study is the largest to date examining racial differences in familiarity, interest, and use of the five ASCO-endorsed IM modalities among patients with breast cancer. Another strength of this study was the inclusion of a racially diverse cohort of patients with breast cancer and key clinical characteristics that previous research was not able to assess.

In conclusion, both Black and White patients with breast cancer were familiar with the five ASCO-endorsed IM modalities, but Black patients expressed greater interest in the use of these modalities. There were no racial differences in prior use of IM, except an increased use of acupuncture among White patients. However, Black patients reported more health care and services access-related barriers than did their White counterparts. Promoting benefits of IM among patients with breast cancer and facilitating patient-provider discussion of IM use may be needed. Furthermore, breast programs focused on health equity should provide access to these services for all patients.

## Data Availability

The data analyzed during the current study are not publicly available due to the ethics for patient information but can be made available from the corresponding author on reasonable request.
